# Supplementary material for: Blocked training facilitates learning of multiple schemas
Source: Commun Psychol. 2024 Apr 9;2:28. doi: 10.1038/s44271-024-00079-4 (PMC11332129; doi:10.1038/s44271-024-00079-4)
Supplement: Supplementary file 1 — Supplementary Material [file 44271_2024_79_MOESM1_ESM.pdf]

Supplementary Information: Blocked training facilitates learning of multiple schemas

Andre O. Beukers[1], Silvy H.P. Collin[2], Ross P. Kempner[1], Nicholas T. Franklin[3], Samuel J. Gershman[3], and Kenneth A. Norman[1]

[1]Department of Psychology and Princeton Neuroscience Institute, Princeton University, Princeton, NJ, USA

[2] Tilburg School of Humanities and Digital Sciences, Tilburg University, Tilburg, The Netherlands

[3] Department of Psychology and Center for Brain Science, Harvard University, Cambridge, MA, USA

Supplementary Information: Blocked training facilitates learning of multiple schemas

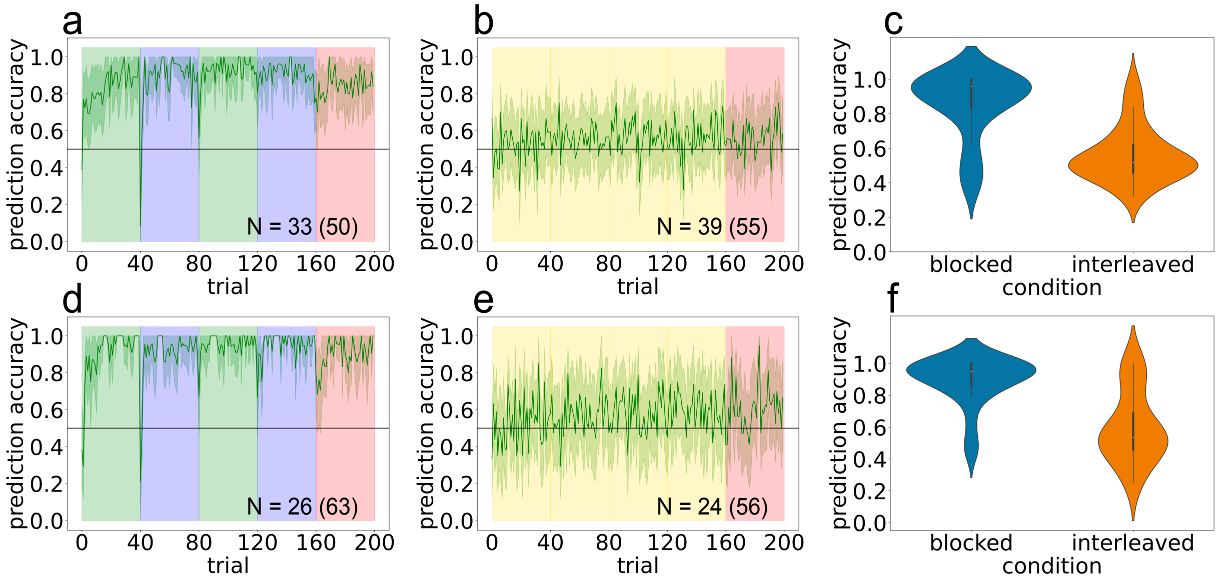

Supplementary Figure 1

Analysis of first of two transition predictions. Note from the graph in Figure 1 that there are two transitions that are (in principle) predictable: from states 3 or 4 to states 5 or 6, and from states 5 or 6 to states 7 or 8. Here we look at the participants' accuracy in predicting the transition from states 3 or 4 to states 5 or 6. (a-c) Initial results (d-f) replicated in independent sample of participants. Left column (a, d) shows blocked results; middle column (b, e) shows interleaved results. (a,b,d,e) Mean accuracy across participants over time. Y-axis is between participant average accuracy, x-axis is time (200 stories). Background colors indicate training curriculum: green is training on chain A only, blue is training on chain B only, yellow is interleaved training, and red is the random-curriculum test phase (50% chance of chain A, 50% chance of chain B). N indicates the number of participants included in the final analysis; the number in parentheses indicates the total number of participants before exclusion (see attention check and exclusion criteria in Methods). Error ribbons indicate ± 1 standard error. (c,f) Violin plots showing accuracy distribution during test phase.

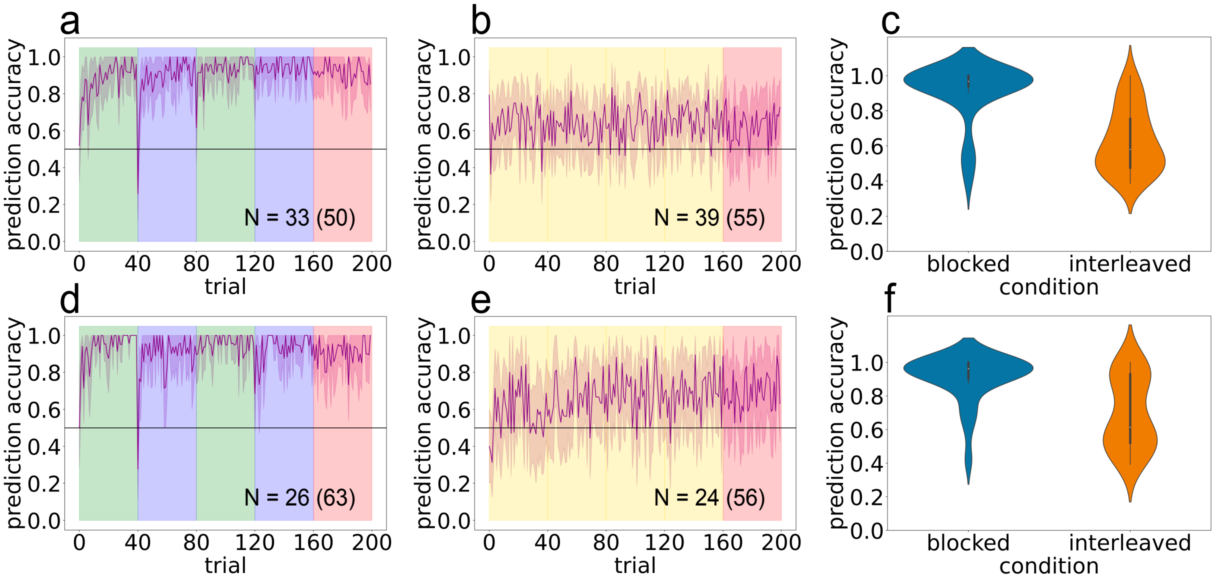

Supplementary Figure 2

Analysis of second of two transition predictions. Note from the graph in Figure 1 that there are two transitions that are (in principle) predictable: from states 3 or 4 to states 5 or 6, and from states 5 or 6 to states 7 or 8. Here we look at the participants' accuracy in predicting the transition from states 5 or 6 to states 7 or 8. (a-c) Initial results (d-f) replicated in independent sample of participants. Left column (a, d) shows blocked results; middle column (b, e) shows interleaved results. (a,b,d,e) Mean accuracy across participants over time. Y-axis is between participant average accuracy, x-axis is time (200 stories). Background colors indicate training curriculum: green is training on chain A only, blue is training on chain B only, yellow is interleaved training, and red is the random-curriculum test phase (50% chance of chain A, 50% chance of chain B). N indicates the number of participants included in the final analysis; the number in parentheses indicates the total number of participants before exclusion (see attention check and exclusion criteria in Methods). Error ribbons indicate ± 1 standard error. (c,f) Violin plots showing accuracy distribution during test phase.

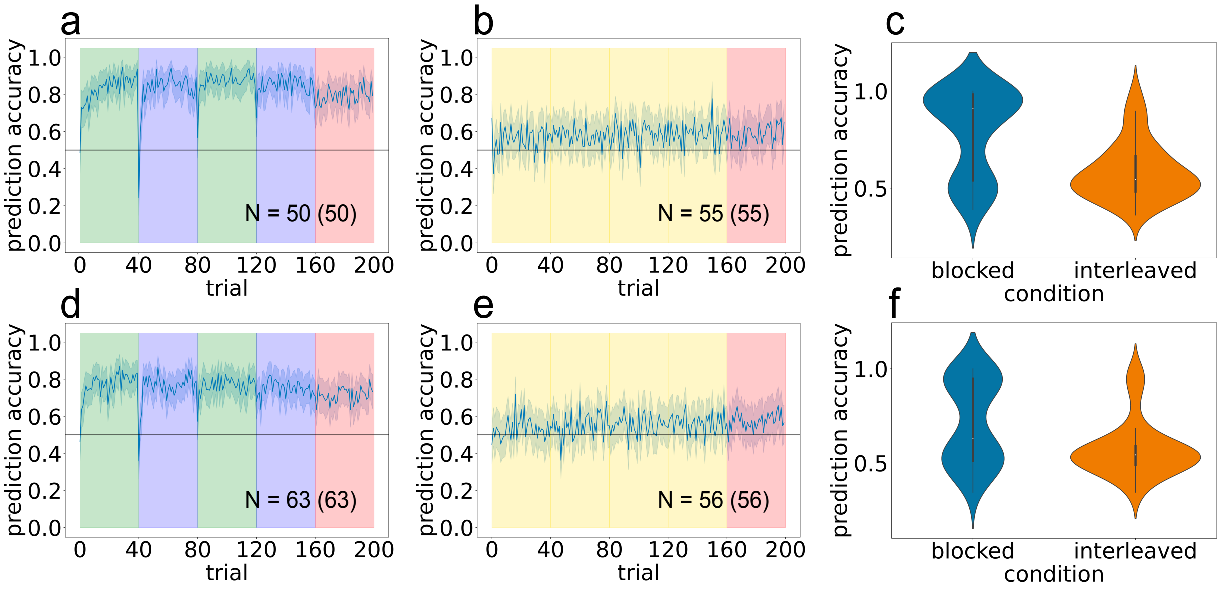

Supplementary Figure 3

Results from blocked and interleaved conditions when all participants are included, regardless of their score on the attention check questions. (a-c) Initial results (d-f) replicated in independent sample of participants. Left column (a, d) shows blocked results; middle column (b, e) shows interleaved results. (a,b,d,e) Mean accuracy across participants over time. Y-axis is between participant average accuracy, x-axis is time (200 stories). Background colors indicate training curriculum: green is training on chain A only, blue is training on chain B only, yellow is interleaved training, and red is the random-curriculum test phase (50% chain A, 50% chain B). N indicates the number of participants included in this analysis, which here equals the total number of participants (shown in parentheses). Error ribbons indicate ± 1 standard error. (c,f) Violin plots showing accuracy distribution during test phase.

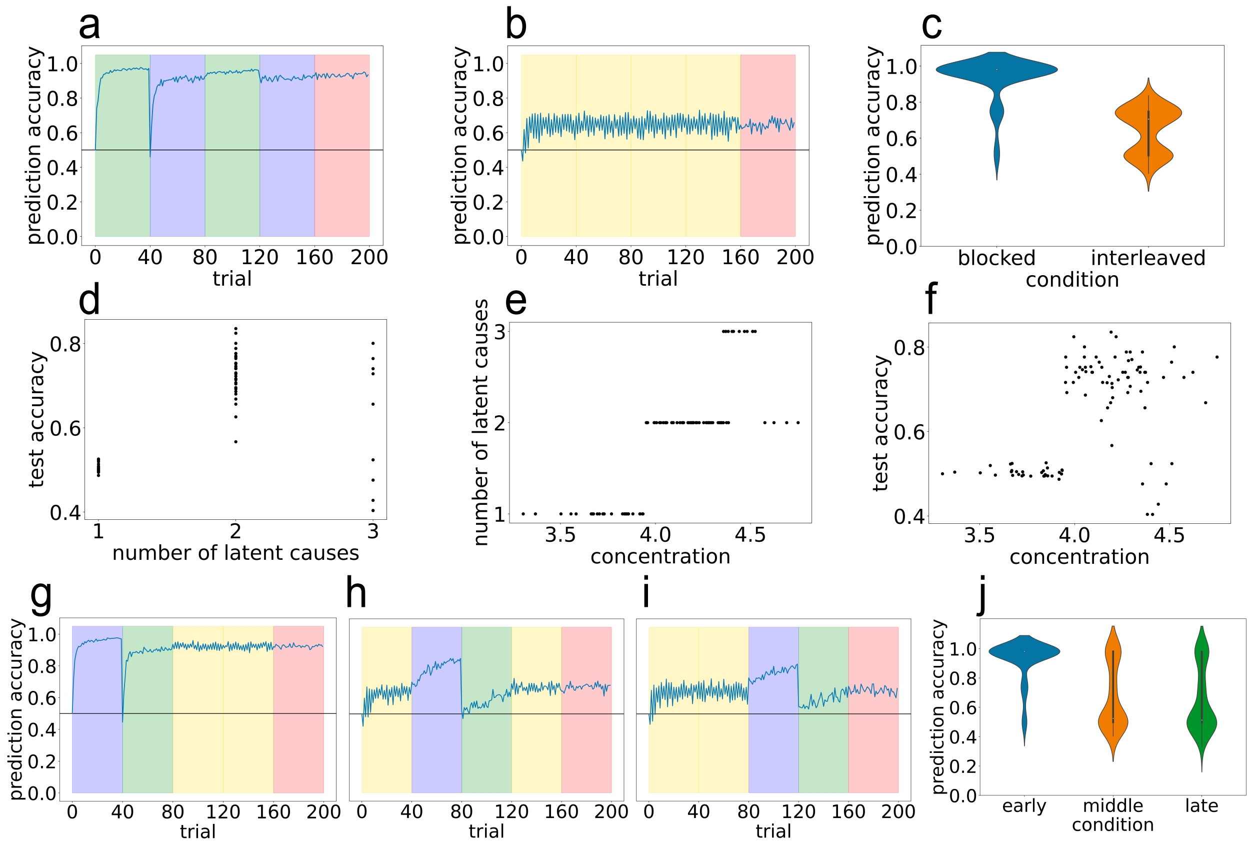

Supplementary Figure 4

Results from a variant of Simulation 3 (compare to Figures 9 and 10) where we assume that blocked learning is needed to get participants to notice the unpredictability of the 3/4 transition. Here, instead of switching off schema inference at the 3/4 timestep for all trials in all conditions, we only switch off schema inference at this timestep after the first block (so, starting on trial 41 for blocked and early, trial 81 for middle and trial 121 for late, and not at all for interleaved). (a,b) Model accuracy over time on blocked and interleaved curricula, respectively. (c) Violin plot of model test accuracy. (d-f) Increased concentration leads the model to split more regularly in the interleaved condition, which improves performance on average. Each dot represents a model run using a slightly different concentration parameter. (d) Number of latent causes versus test accuracy; (e) concentration versus number of latent causes; (f) concentration versus test accuracy. (g-i) Model predictions on early, middle and late curricula, respectively. (j) Violin plots of test accuracy predicted by the model on each curriculum. Best-fit parameters were obtained using a new gridsearch for this model variant. Overall, results are somewhat more complex than in Simulation 3, but the same qualitative patterns are present.

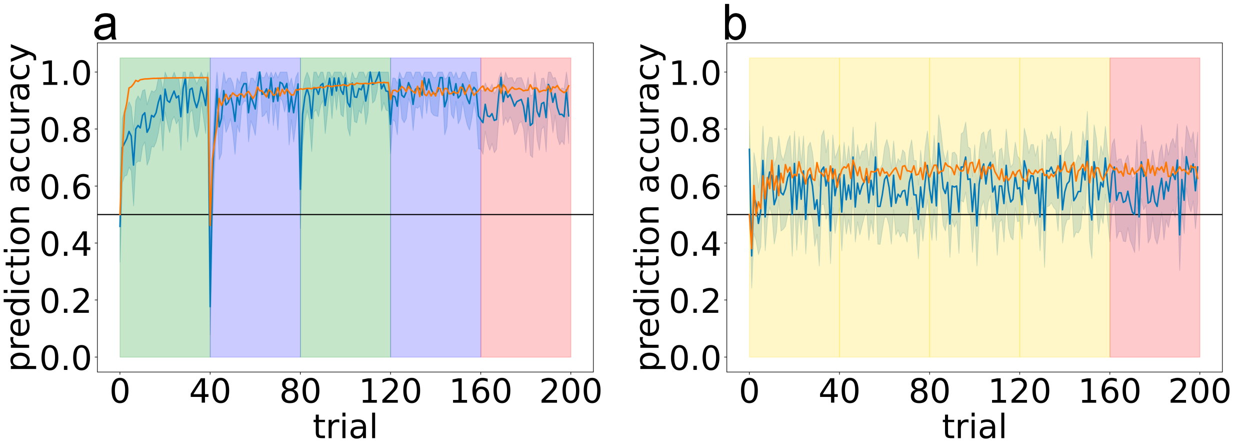

Supplementary Figure 5

Model fits for Simulation 2, overlaid on human data from the original blocked and interleaved experiments.

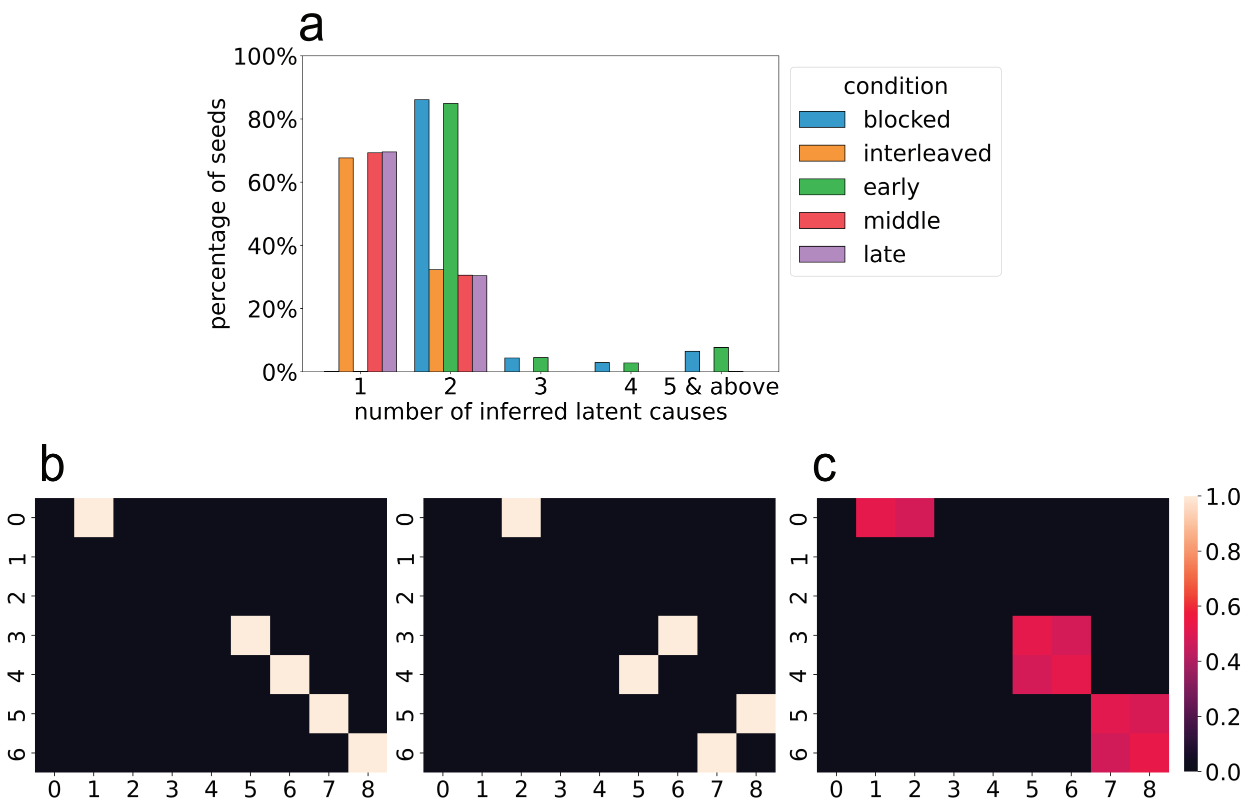

Supplementary Figure 6

Analysis of the number of inferred latent causes and their properties. (a) Histogram of the number of inferred latent causes in each of the model conditions for the model used in Simulation 3. (b) Plot of the learned transition matrices in Simulation 3 in the case where the model infers two latent causes (in either the interleaved or blocked condition), and (c) plot of the learned transition matrix in Simulation 3 in the case where the model only infers one latent cause (in the interleaved condition). When the model infers two latent causes, the learned transition matrices accurately reflect the ground truth transition structure; when the model only infers one latent cause, it learns a transition matrix that averages across the ground-truth transition matrices for the two Markov chains. Note that the rows corresponding to the transition from states 1 and 2 to the following state are empty because this unpredictable transition was not shown to this version of the model (see discussion of this point in Simulation 2, and also in the Limitations section of the Discussion).

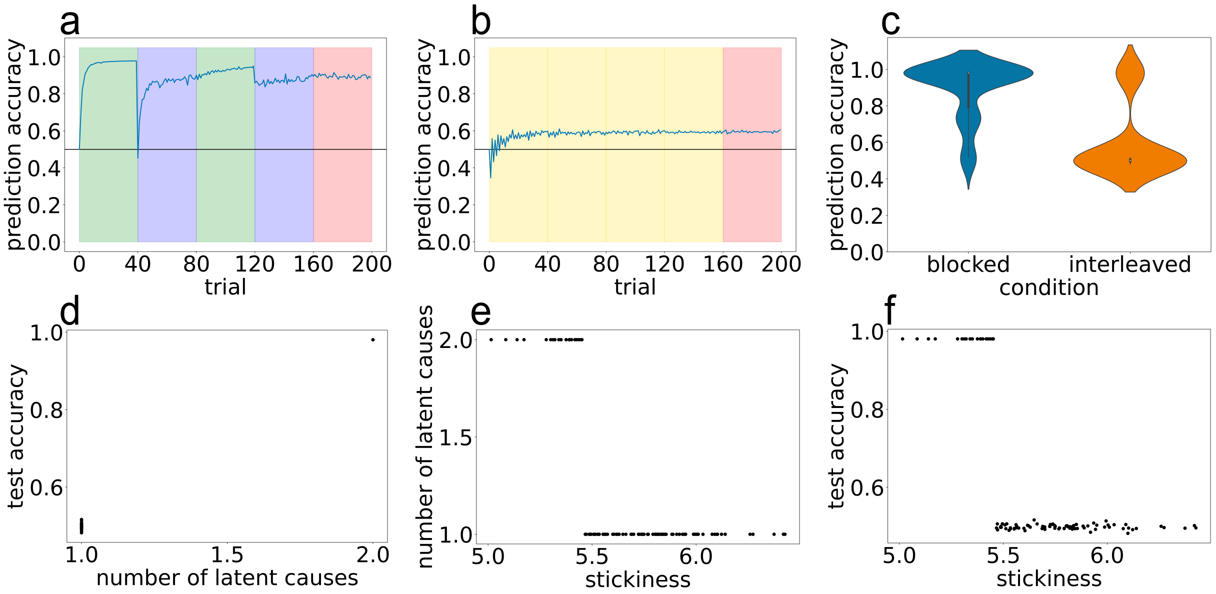

Supplementary Figure 7

Individual differences in model stickiness can potentially explain human performance in the interleaved condition (compare to Figure 9). (a,b) Model accuracy over time on blocked and interleaved curricula, respectively. (c) Violin plot of model test accuracy. (d-f) Reduced stickiness leads the model to split more regularly in the interleaved condition, which improves performance. Each dot represents a model run using a slightly different concentration parameter. (d) Number of latent causes versus test accuracy; (e) stickiness versus number of latent causes; (f) stickiness versus test accuracy.

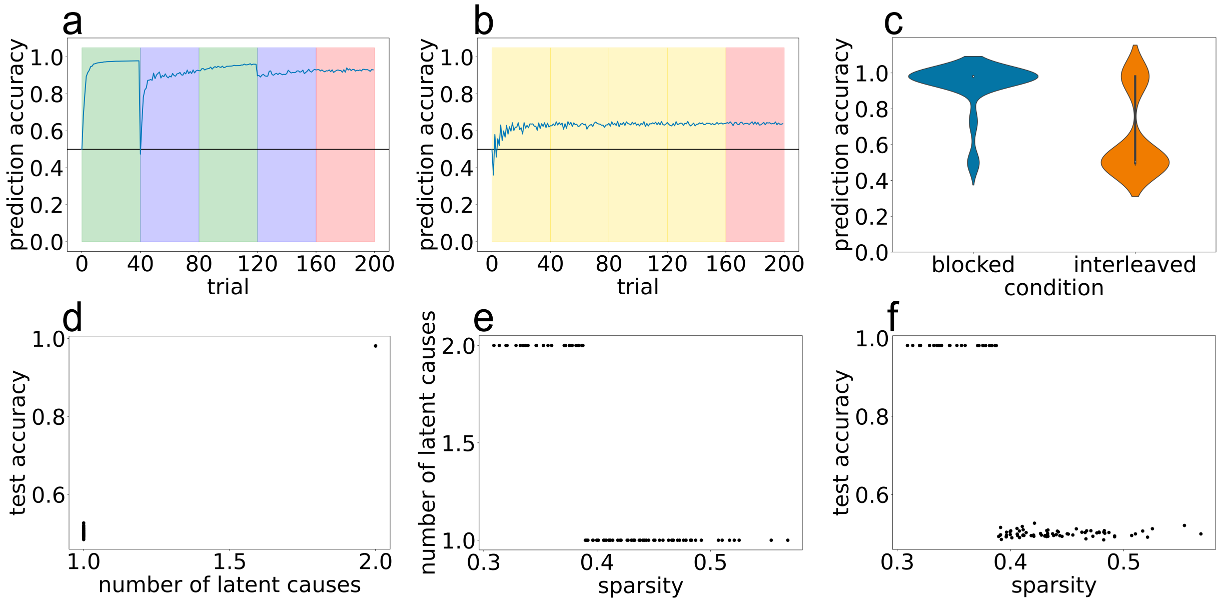

Supplementary Figure 8

Individual differences in model sparsity can potentially explain human performance in the interleaved condition (compare to Figure 9). (a,b) Model accuracy over time on blocked and interleaved curricula, respectively. (c) Violin plot of model test accuracy. (d-f) Reduced sparsity leads the model to split more regularly in the interleaved condition, which improves performance. Each dot represents a model run using a slightly different concentration parameter. (d) Number of latent causes versus test accuracy; (e) sparsity versus number of latent causes; (f) sparsity versus test accuracy.

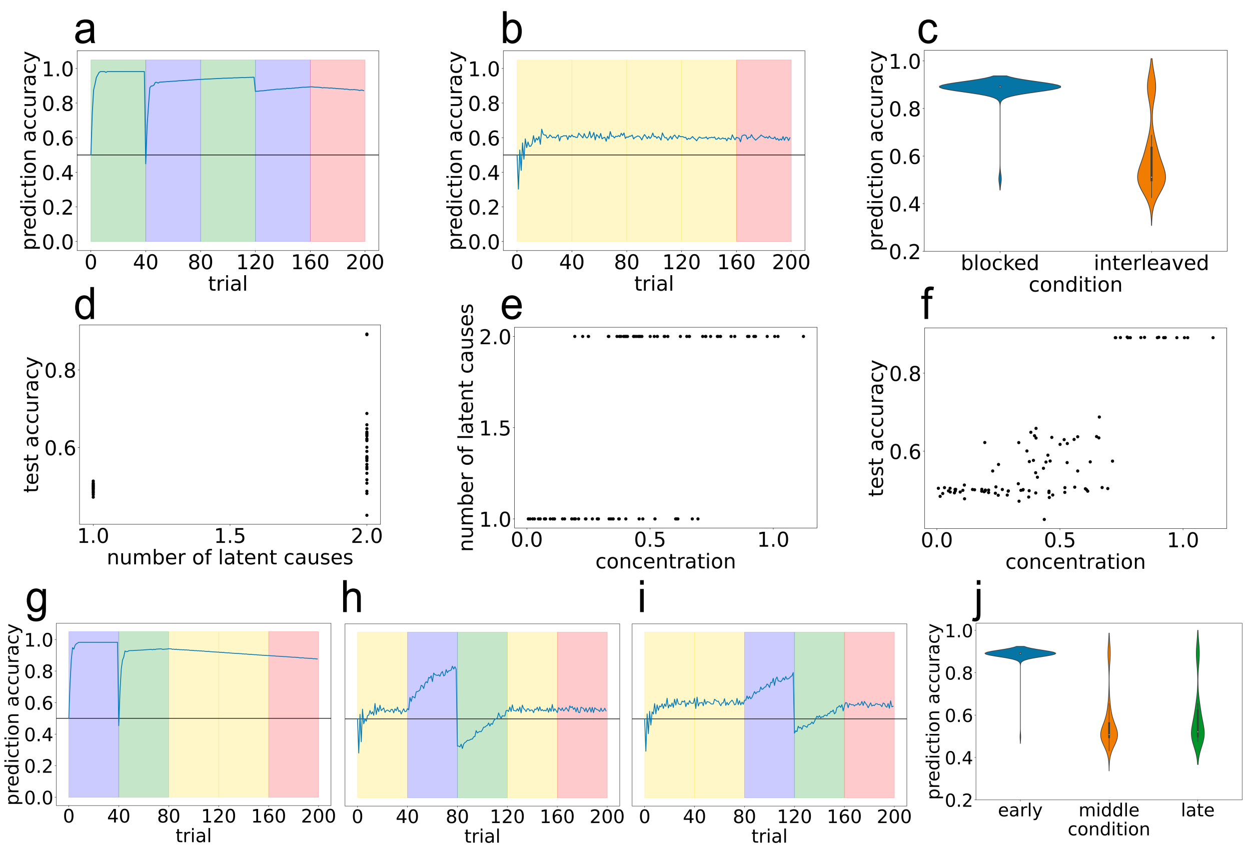

Supplementary Figure 9

Results from a variant of Simulation 3 (compare to Figures 9 and 10) where we use the Bayesian optimal predictive distribution (marginalizing across schemas) rather than our default approximation where only the currently active schema is used to make predictions. (a,b) Model accuracy over time on blocked and interleaved curricula, respectively. (c) Violin plot of model test accuracy. (d-f) Increased concentration leads the model to split more regularly in the interleaved condition, which improves performance on average. Each dot represents a model run using a slightly different concentration parameter. (d) Number of latent causes versus test accuracy; (e) concentration versus number of latent causes; (f) concentration versus test accuracy. (g-i) Model predictions on early, middle and late curricula, respectively. (j) Violin plots of test accuracy predicted by the model on each curriculum. Best-fit parameters were obtained using a new gridsearch for this model variant. Overall, model fits are qualitatively similar to those in Simulation 3, although the fit to human data is quantitatively worse when we use the Bayesian optimal predictive distribution (depicted here) vs. when only the currently active schema is used to make predictions (in Simulation 3). Here, MSE=0.0432, compared to MSE=0.0371 in Simulation 3. This MSE difference corresponds to an AIC difference of 122.12 (AIC for Bayesian optimal approach = -2507.87; AIC for approximation = -2629.96).
